# Primary amputation vs limb salvage in mangled extremity: a systematic review of the current scoring system

**DOI:** 10.1186/s12891-015-0832-7

**Published:** 2015-12-02

**Authors:** Giuseppe Rosario Schirò, Sergio Sessa, Andrea Piccioli, Giulio Maccauro

**Affiliations:** Departement of Orthopaedic Science and Traumatology, Catholic University Rome, Rome, Italy; Palazzo Baleani, Umberto I Hospital Rome, Rome, Italy; Galeazzi Orthopaedic Hospital, Milan, Italy; Niguarda Cà Granda Hospital, Piazza Ospedale Maggiore 3, 20162 Milan, Italy

## Abstract

**Background:**

In the last decades a lot of new reconstructive techniques were developed for the treatment of mangled lower extremity. However failed attempt to limb salvage is related to high risk of mortality for the patient. Several scores were developed to establish guidelines for the decision to amputate or not, however in literature there is no consensus about the reliability of this scores.

**Methods:**

The authors focused their attention on the most used score system to provide guidance of the management of a mangled lower limb. The search term used included mangled lower extremity, MESS, PSI, LSI and NISSSA. The inclusion criteria were: studies dealing with mangled lower extremity; articles reporting MESS, PSI, LSI or NISSSA scores; articles published in English in PubMed, Cochrane, Scopus and web of science in the last 30 years, minimum number of cases in study of 15, minimum follow up of 1 year.

**Results:**

According with the criteria described above, we found 134 articles in PubMed, 165 articles in Scopus, 111 articles in the Cochrane Library and 108 articles in Web of Science. The most used score in literature is the MESS. Few results are shown using the other severity scores. There are a lot of controversies in literature about the use of this scale. MESS seems to be more accurate than the LSI in prediction of limb salvage. LSI score shows better results when applied to type III tibial fractures. High sentivity of the PSI score is described when applied to predict successful limb salvage. Low sensitivity and specificity are described for the NISSSA score. The literature is very poor of articles related to mangled lower extremity in children. Higher sensitivity and specificity are described for these scores in children when compared to adult population.

**Conclusion:**

The mangled lower extremity treatment is a challenge for the surgeon. Many scores were developed to help the surgeon, however they cannot be used as the sole criterion by which amputation decision are made and, in case of succesful limb salvage, they are not predictive of the functional recovery. Moreover, undue enthusiasm for new surgical techniques can lead to increased morbidity and mortality in case of secondary amputation.

## Background

High lower extremity trauma is a challenge for the surgeons. A lot of patients with severe mangled extremities are young working people. New reconstructive techniques allow trying limb salvage also in complex lesions that could be treated only by amputation in the past decades. However, failed attempt to limb salvage is associated with increase in morbidity and mortality. The topic to try a limb salvage or to perform a primary amputation remains a big issue. Several authors proposed different types of scores to classify the severity of lesions and to establish guidelines regarding the decision to amputate or not [1–4]. Most common lower-extremity injury-severity scoring systems include the Mangled Extremity Severity Score (MESS) that analyzes soft tissues injury, limb ischemia, presence and duration of shock and the age of patient [2]; the Predictive Salvage Index (PSI) that focuses the attention on the warm ischemia, the bone and muscles damage and on the extent of vascular injury [1]; the Limb Salvage Index (LSI) considering artery, nerve, bone, skin, muscles and warm ischemia time [4]; the Nerve Injury, Ischemia, Soft-Tissue Injury, Skeletal Injury, Shock and Age of Patient (NISSSA) Score [3]. MESS score is probably the most common scoring system used [5]. In literature there is no consensus about the reliability to predict functional outcome and secondary amputation of these scores. Principle variables considered by the developers are: soft tissue, time of ischemia, bone and nerve injury, blood loss (Table 1) [6]. Bosse et al. [7] evaluated prospectively 556 high energy lower extremity injuries with the use of data collected as a part of a multicenter study (LEAP: Lower Extremity Assessment Project) developed to compare clinical outcome of lower mangled limb after salvage or amputation; the Authors did not support the utility of any of these scores to make a decision to amputate or not. In their study the authors described different specifity and sensibility for each of these scores: regarding PSI the sensitivity and specificity were 56 and 79 % considering ischemic limb injury; while the sensitivity of the MESS was 46 % considering all limbs and 72 % if only ischemic limb were considered, this score demostred high specificity to predict limbs amputation. NISSSA score were more sensitive than MESS score (81,8 %) and the value of the specificity was 92,3 %. LSI score demostred a specificity of 82 % and sensitivity 83 % considering ischemic limbs. Only little data exist about the effectiveness of these scores in children, however some authors affirm that there is more sensitivity in child population compared with adult population [5, 6, 8]. Moreover some studies demon strate that in the case of more severe lesions the long term outcome is the same in the patients that underwent reconstructive surgery and in those that underwent amputation, with more complications and economic impact for the first group [9–11]. The goals of this study is to analyze the most used scoring systems described in literature for the assessment of mangled extremity to provide guidance of the management of a mangled lower limb and propose a summary of the common controversies and clinical data available.

**Table 1:** Variables considered in each score

	MESS	LSI	PSI	NISSSA
Age	x			x
Shock	x			x
Worm Ischemia time	x	x	x	x
Bone injury		x	x	
Muscle injury		x	x	
Skin injury		x		
Nerve injury		x		x
Deep vein injury		x		
Skeletal/soft tissue injury	x			x
Contamination				x
Time to treatment		x		
Co-morbid condition				

## Methods

The authors used the PRISMA checklist to reduce the incidence of bias.

The inclusion criteria were: studies dealing with mangled lower extremity; articles reporting MESS, PSI, LSI or NISSSA scores; articles published in English in PubMed, Cochrane, Scopus and web of science in the last 30 years, minimum number of cases in study of 15, minimum follow up of 1 year. Exclusion criteria were: non-English studies, case report and previous leg or foot amputation.

All eligible studies underwent quality scoring that consisted of answering some questions:

- Are the inclusion–exclusion criteria defined?
- Are the outcome critera well defined?
- Is the type of treatment adequate and well described?
- Is it a prospective study?
- Is the mean follow up more than 2 years?

For each question an affirmative answer scored 2 points, a negative answer scored 0 points. Only study with more than 6 points were considered.

Data of each article were considered independently.

The search term used included mangled lower extremity; articles reporting MESS, PSI, LSI, NISSSA with the appropriate Boolean linkage terms, for example “And”, “Or”,“Not”, etc.

## Results

**Fig. 1:**
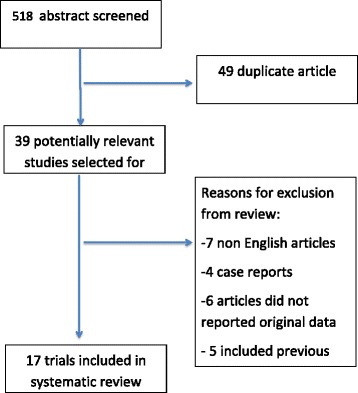
Showing the search algorithm

According with the criteria described above, we found 134 articles in PubMed, 165 articles in Scopus, 111 articles in the Cochrane Library and 108 articles in Web of Science (Fig. 1). The most used score in literature is the MESS, for example combining “mangled lower extremity” and “MESS” we found 45 results in PubMed, only 6 results are shown combining “mangled lower extremity” and “LSI”. Few results are shown using the other severity scores.

The sensitivity was defined as the probability that limbs requiring an amputation will have limb-salvage scores at or above the index threshold, specificity was defined as the probability that salvaged limbs will have limb-salvage scores below the threshold.

### MESS

The Mess score [2] was developed in 1990 on a retrospective analysis of 25 patients and subsequently on a prospective study on 26 patients. The main evaluated variables are: soft tissues injury, limb ischemia, presence and duration of shock and the age of patient. A MESS of >7 points predicted amputation (Table 2). There is a lot of controversy in literature about the use of this scale. Elsharawy et al [12] in his prospective study considered 46 upper and lower extremities and described, considering MESS injuries scoring higher than 7 and only secondary amputation, a MESS score specificity of 27.5 %. Menakuru [13] in his study confirmed the results obtained by Elsharawy et al. He was able to save the limb in 20 (69 %) of 29 patients with MESS higher than 7. However, in literature there are some reports, which present different results about this score and describe a good correlation between MESS higher than 7 and amputation. [14, 15] For example Sharma [16] and Korompilias [17] amputated all limbs with MESS higher than 7 reporting good results. The percentage of primary amputation for MESS > 7 vary from 0 to 41 %. Korompilias [17] considered 10 cases of massive extremity injuries. He tried to save the limbs. The results were that three patients died and the others were amputated within 15 days of initial salvage. Durham [18] considered 51 limbs; 21 had a MESS higher than 7. The percentage of primary amputation was 41,1 % while the percentage of secondary amputation was 11,7 %. In their works authors described a sensitivity of 79 % and a sensibility of 83 % of the MESS in prediction of limb salvage. The percentage of secondary amputation vary from 1,8 % [19] to 15,6 % [20]. O’Sullivan et al. [14] analyzed 54 mangled lower limb and affirmed that MESS was more accurate than the LSI in prediction of limb salvage. A MESS score of > 7 offered a greater relative risk of amputation (9.2) than an LSI score of > 6 (5.3). There are some studies regarding mangled lower extremities in combat situation. They found a correlation between MESS higher than 7 and amputation. Rush et al. [15] recognize the utility of the MESS score in a study in which he considered 60 limb injuries in Iraq an Afghanistan war. Brown [21] considered 77 military patients with 86 limb injuries from Iraq and Afganistan war. He was able to save 74 % of limbs while 26 % were amputated (18 % underwent primary amputation and 8 % secondary amputation). For this reason the Authors affirmed that MESS score does not allow us to decide whether or not to amputate. In combat situation the percentage of primary amputation vary from 3,1 % [22] to 17,4 % [23]. The largest study reporting lower extremity injury severity score was reported by Bosse [7]. He considered 556 lower extremities by using the main scoring systems. He found that even though 14.5 % of patients had a MESS score < 7 they still underwent amputation. The authors found that LSI had better specificity than PSI, MESS and NISSSA. The MESS had 69.9 % specificity and 78 % sensitivity. O’Sullivan [14] in his study considering Gustillo IIIB and C injuries concluded that MESS and LSI weren’t predictive for amputation (Table 3).

**Table 2:** MESS Absolute indication for amputation: >7

Energy	Low	1
	Medium	2
	High	3
	very high	4
Ischemia	Perfused	1
	Pulse absent	2
	Cool, paralyzed,insensate	3
Shock	SBP > 90	0
	Transient	1
	Hypotension persistent hypotension	2
Age (years)	<30	0
	30–50	1
	>50	2

**Table 3:** Results for MESS > 7

Authors	Limbs	Amputation	Salvaged
Brawn	86	18	35,71 %
Bosse	556	68	34,6 %
Elsharawy	62	3	93,4 %
Korompilas	63	7	0 %
Kumar	61	10	9,09 %
Menakuru	148	9	68,9 %

### LSI

The LSI [4] score was developed in 1991. The variables about injury considered in this score are: artery, nerve, bone, skin, muscles and warm ischemia time. Each scoring system has a threshold value. If the total score exceeds the critical point early amputation should be considered. An LSI of >6 points indicates that the limb should be amputated (Table 4). The score was developed on the bases of retrospective studies on small group of patients. The authors reported 100 % correlation between the limb outcome and the threshold score. Bosse et al. [7] reported different values. LSI showed better performance then other scores especially when applied to type III tibial fractures. When applied to the ischemic limbs LSI showed a sensitivity of 83 % and a specificity of 82 %. O’ Sullivan et al. [14] found that LSI was more accurate in predicting amputation when the limbs which required delayed amputation were analysed, compared with MESS. In his work Dhuram [18] described a sensitivity an a specificity of 83 % in prediction of limb salavage.

**Table 4:** Absolute indication for amputation: LSI > 6 or Gustillo IIIC with nerve injury

Artery	0–2
Nerve	0–2
Bone	0–2
Skin	0–1
Muscle	0–2
Deep vein	0–1
Warm ischemia time	0–4

### PSI

The PSI [1] was proposed by Howe in the 1987, the study was a retrospective analysis of 21 patient and focuses the attention on the warm ischemia, the bone and muscles damage and on the extent of vascular injury. He threshold for limb amputation is a score of >8 points (Table 5). The authors reported a sensitivity of 78 % and a specificity of 100 %. Bosse et al. [7] analyzed 556 ischemic limb injuries and found a sensitivity and specificity of 56 and 79 % when immediate amputations were included in the analysis and 40 and 79 % when immediate amputations were excluded. No better results were described when only open tibial fractures were considered. Dhuram [18], on the other hand, described a very high sensitivity (96 %) and a very low sensibility (50 %) when this score is used to predict successful limb salvage.

**Table 5:** PSI. Absolute indication for amputation: >8

Artery	1–3
Time to surgery	1–3
Bone	1–3
Muscle	1–3

### NISSSA

The NISSSA score was proposed by McNamara et al. [3] in 1994. The authors focused their attention on the nerve injury because in their opinion a loss of plantar sensation is a crucial component to make an amputation. The threshold for limb amputation is a score of >11 points (Table 6). This score was developed on a retrospective analysis of 26 patients. The NISSSA score was found to very sensitive (81.8 %) and specific (92.3 %). However in their study Bosse et al [7] described different findings: the NISSSA had a sensitivity of 33 % when applied to all type-III tibial fractures and of 13 % when immediate amputations were excluded.

**Table 6:** NISSSA. Absolute indication for amputation: >11

Nerve	UP TO 3
Ischemia	UP TO 6
soft tissue	UP TO 3
Skeletal injury	UP TO 3
Shock	UP TO 2
Age	UP TO 2

### Scores in children

The literature is very poor of articles related to mangled lower extremity in children. All the examined articles reported good prognostic power of the MESS in this group. In his study on 26 children, Stewart et al. [24] described a sensitivity of the MESS of 100 % and specificity of 87 % when applied to patients with tibial trauma only. The Authors found higher sensitivity for all scoring system when compared to the analysis by Bosse at al in adult population. Behdad et al. [20] evaluated 200 children with lower extremity long bone open fracture using MESS score, the Authors described a sensitivity of 73 % and a specificity of 54 % for a MESS ≥ 6.5. Fagelman et al [25] described an accurate prediction of 93 % when MESS is applied to grade III lower extremity fracture in children. Mommsen et al. [26] analyzed 27 lower extremities in children. In all patients with a MESS < 7 the lower extremity was salvaged. In contrast, patients with a MESS ≥ 7 of the lower extremity underwent initial amputation in 25 %. A definite salvage of the lower extremity was achieved in 33.3 % (Table 7).

**Table 7:** MESS specificity and sensitivity in children

Author	Specificity	Sensitivity
Stewart	87 %	100 %
Behdad	54 %	73 %
Mommsen	67 %	100 %
Fagelmann	93 %	63 %

## Discussion

The decision to try limb salvage or to amputate in case of lower mangled extremity is a challenge for the clinicians. In most cases the treatment should not be decided on the basis of the first evaluation. A wrong decision to try limb salvage will result in a secondary amputation and will subject the patient to great physical, psychological, financial and social sufferings [27]. Failed effort to save a limb can results in more hospital cost and increased patient mortality [7]. In the last years a lot of new techniques to try mangled limb salvage were developed. Much attention should be given to the use of these new techniques, as described by Dirschi and Daners [28], the growing enthusiasm for microvascular surgery may lead prolonged unsuccessful attempts at salvage and subsequently to death, sepsis and preservation of dysfunctional limbs, as well as higher adjusted hospital charges. Other authors pointed their attention on the topic of secondary amputation. Bondurant et al. [9] analyzed mortality, number of procedures, hospital stay and cost in primary versus secondary amputation on a cohort of 43 patients that underwent amputation for grade III open tibia fracture. The secondary amputation group showed a 21 % of mortality, significant increase in day of hospitalization, cost and number of surgical procedures. Some studies have shown that primary amputation (8-26) is associated to worse functional outcome compared to delayed amputation. Alexander Bee Dagum et al. [29] analyzed retrospectively 55 severe lower extremity injuries, 46 of them underwent attempted salvage, 11 % of them required secondary amputation. They found no predictive power of the mangled limb scoring system and concluded that they didn’t add any contribute to surgeon’s decision making. As described by Fodor et al. [5], failed attempts at limb salvage result in prolonged hospitalization, along with multiple surgical procedures, pain and psychological trauma. However salvaged limb does not guarantee functionality, normal life, a pain-free extremity or employability [5]. Akula et al. [30] performed a meta-analysis to evaluate the quality of life in patients that underwent post-traumatic amputees compared with those that underwent limb salvage. They found that reconstruction is more psychologically acceptable for the patient than amputation, while the physical outcome was the same for both the treatments. In their work, Hitmann et al. [31] concluded that technical viability is not a sufficient criterion for limb salvage. In 2007 Bosse et al. [11] performed a meta-analysis on 9 observational studies in order to give an answer to the difficult answer: whether to perform primary amputation? They described really interesting results. Hospital stay was similar for both treatment, but salvage group had longer rehab and higher cost with more complication rate. Return to work and long term functional results were similar. It is crucial for the patient to realize that amputation doesn’t reflect a failure of the treatment, but it’s the first step for rehabilitation [32]. Moreover, the patient of the second group required more surgical procedures than those of the first group (6,9 vs 1,6). Some authors focused on the economical impact of different kind of treatment. Bondurant et al. [9] analyzed 263 patients with open tibia fracture, 14 of them underwent primary amputation, 29 of the underwent delayed amputation. The authors described twice the number of days of hospitalization for the second group of patients and higher hospital costs (53,4 days vs 22,3 days and 53,462 dollars vs $28,964 dollars). Georgiadis et al. [33] compared 16 patients with successful limb salvage and 18 with early amputation, they showed higher hospital charges for the group of limb salvage: 65,624 vs 109,044$.

In literature the main variables evaluated to chose the type of treatment after severe lower limb trauma are: soft tissue health, time of ischemia, blood loss, bone status and nerve injury [8]. In authors opinion, neurovascular lesions and soft tissue defects represent the main prognostic factor determining the fate of the limb. Several authors developed different scores to help the surgeons in the difficult decision to amputate or not a mangled lower limb. More used scores in literature are MESS [2], PSI [1], LSI [4], NISSSA [3]. However these scores were developed since 15 years ago and in the last years a lot of new surgical procedures were developed. Several studies in literature analyzed the reliability of these scores in the decision to amputate or not. In the 2008 Ly et al. [34] published a prospective Level I study on a cohort of 601 patients. They restricted the study at 407 salvaged limbs and concluded that lower extremity injury severity scoring systems are not predictive of functional recovery among patients who undergone successful limb reconstructions. In their study, Bonanni et al [35] scored retrospectively 58 limb salvage attempts. In their study none of the analyzed scoring systems showed predictive utility. Patients with bilateral mangled lower limb represent a challenge for the surgeon. The LEAP study analyzed separately this group of lesions. A total of 32 bilateral injuries were analyzed, 13 had bilateral salvage, 10 had bilateral amputation and 8 had unilateral amputation. Patients who underwent unilateral salvage/amputation showed the best return to work rate. Higher complication rate was found in bilateral salvage group than bilateral amputation group. However, the authors concluded that disability for bilateral mangled limb is no more than the unilateral group [36]. There are only few studies in literature about the utility of the predictive scores in children. In child population soft tissue have better healing capacity than adults and periostium in children have great capacity to form bone [37]. In Authors opinion it is better, at first time, to try limb salvage in children also in presence of high severity score. Elsharawy et al. [12] described a case of limb salvage in a child with clinical picture of irreversible ischemia and high mangled severity scores. They recommended trying to revascularize and to save the limb in children, regardless clinical condition and severity scores. The lower extremity injury scoring system seems to have good predictive capacity in child population. In literature all the studies reported good correlation between MESS and limb salvage or amputation [5].

In this analysis of the literature none of the analyzed score shows reliability for discriminating the limb requiring primary or secondary amputation or to predict functional outcome after successful limb salvage. In literature the lower injury severity scores lack sensitivity. However the analyzed works shows how in some cases limb salvage is associated to worst functional results than primary amputation. MESS is the most used scoring system followed by the LSI, however there is no consensus in literature about specificity and sensibility of this score. The MESS is less complex than LSI to apply, and unlike the LSI, enjoys the advantage that may be determined preoperatively. PSI seems to be more usefull to predict raccomanded amputation in most severe injuries due to its high sensitivity and low specificity, a great advantage of this score is its semplicity to be used, on the contrary soft tissue are not well considered. NISSSA has the advantage to include assessment of the nerve injury, however it is little used probably due to its low sensitivity and specificity. Only few data exist about reliability of the other analyzed score. For this reasons the future of a limb should not be decided on the basis of initial evaluation and the lower extremity severity scoring system have limited usefulness. In literature, delayed amputation and limb salvage procedures are associated with higher hospital charges, more surgical procedures and higher hospitalization days than early amputation [9]. This study has some limitations. The methodologic quality of the analyzed trials was not homogenous. However the Authors excluded case reports and case series with low methodological quality. An experienced and dedicated team of surgeons is crucial to obtain successfull limb salvage. The Authors in this review analyzed trials occurred in trauma centres of different levels: the results obtained in the trials included in this review are related to the level of the hospital in which the limbs were treated.

## Conclusion

Finally, in the last years, a lot of new techniques to try limb salvage were developed. Collaboration between orthopedic surgeon, plastic surgeon and vascular surgeon is very important for a good treatment. However, undue enthusiasm for microvascular surgery and others new techniques can lead to increased mortality and morbidity with longer hospitalization and higher cost. Moreover, in some cases the salvaged limb does not guarantee a good functionality. On the other hand, child population has a great healing capacity and, in Authors opinion, it’s better to attempt limb salvage. The most used scoring systems are not useful to predict functional outcome of the lower mangled extremity and to decide to amputate or salvage a limb. In Authors opinion severity scoring systems analyzed are not predictive of functional recovery in patients who have undergone successful limb reconstruction and they could be considered only as an help deciding to amputate or not a mangled lower limb. Surgeons should exercise caution when interpreting scores, their experience is a crucial point for the decision.
